# An Innovative Approach Based on the Green Synthesis of Silver Nanoparticles Using Pomegranate Peel Extract for Antibacterial Purposes

**DOI:** 10.1155/bca/2009069

**Published:** 2025-03-13

**Authors:** Rocío Díaz-Puertas, Francisco J. Álvarez-Martínez, Enrique Rodríguez-Cañas, Fernando Borrás, Artur J. M. Valente, José A. Paixao, Alberto Falcó, Ricardo Mallavia

**Affiliations:** ^1^Institute of Research, Development and Innovation in Healthcare Biotechnology in Elche (IDiBE), Miguel Hernández University, Elche 03202, Alicante, Spain; ^2^Statistics and Operative Research Department, Miguel Hernández University, Elche 03202, Alicante, Spain; ^3^Department of Chemistry, University of Coimbra, Coimbra 3004-535, Portugal; ^4^Department of Physics, University of Coimbra, Coimbra 3004-516, Portugal; ^5^Fish Pathology Group, Institute of Aquaculture Torre de La Sal-Spanish National Research Council (IATS-CSIC), Cabanes 12595, Castellón, Spain

**Keywords:** antibacterial activity, Box–Behnken, green synthesis, *Punica granatum*, response surface methodology, silver nanoparticles

## Abstract

This study describes a green synthesis method for silver nanoparticles (AgNPs) using autochthonous “Mollar de Elche” pomegranate peel extract and optimized through a Python-programmed Box–Behnken design (BBD) created specifically for the work. The bioactive compounds in pomegranate, particularly punicalagin, serve as effective reducing and stabilizing agents. BBD was used to analyze the effects of dependent variables such as silver nitrate concentration, pomegranate extract concentration, and temperature on responses such as hydrodynamic diameter, polydispersity index, and zeta potential, minimizing experimental trials and highlighting variable interactions. Optimal conditions were experimentally validated and agreed well with the predicted values. The optimized AgNPs were characterized via ultraviolet-visible spectrophotometry, Fourier transform infrared spectroscopy, X-ray diffraction, and field emission scanning electron microscopy. These AgNPs demonstrated substantial antibacterial activity against *Escherichia coli* and *Staphylococcus aureus*. Furthermore, the AgNPs were incorporated into nanofibrous scaffolds as a proof of concept for potential biomedical applications, where their antibacterial activity was partially retained postincorporation. This study highlights the potential of pomegranate extract as a sustainable medium for AgNP synthesis with promising antibacterial applications and the ability of the BBD as a useful tool for efficient optimization of multivariable processes, including the synthesis of nanomaterials.

## 1. Introduction

Metal nanoparticles (MNPs) play an essential role in the area of nanotechnology and material science due to their excellent physicochemical properties, such as remarkable mechanical and thermal stability, high surface area, and notable optical and magnetic properties [1]. These distinctive properties make MNPs highly valuable in diverse applications across various fields, including catalysis, medicine, electronics, and environmental remediation [2]. In particular, silver NPs (AgNPs) have garnered considerable scientific interest, primarily due to their unique chemical, physical, and biological characteristics. In particular, AgNPs have demonstrated substantial potential in biocidal and antimicrobial applications, exhibiting efficacy against both Gram-negative and Gram-positive bacterial strains [3].

AgNPs can be synthesized through physical, chemical, and biological approaches. While physical and chemical methods yield high-purity AgNPs, their drawbacks, such as high energy consumption and the use of toxic agents, restrict their applicability [4]. The pursuit of innovative AgNP synthesis now focuses on improving quality and properties while prioritizing environmental responsibility. In this context, “green” or biological synthesis has emerged as a promising sustainable alternative to traditional methods [5]. This environmentally friendly route uses microbial, plant-, fungi-, or algae-derived products as reducing and capping agents [6] and offers several advantages, including waste reduction, the utilization of safer solvents and renewable feedstock, simplicity, and cost-effectiveness [7].

The use of plant extracts/molecules in the green synthesis of AgNPs offers additional advantages to produce NPs with precise dimensions, morphology, and composition, including less hazardous procedures and a vast repertoire of biomolecules [8]. These extracts contain a diverse array of bioactive compounds, including proteins, amino acids, polysaccharides, terpenes, alkaloids, phenolics, saponins, and vitamins [9]. This diverse set of biomolecules acts as highly effective reducing and capping agents, playing a central role in the transformation of Ag ions to assemble AgNPs [10]. Such compounds may enable the synthesis of AgNPs with unique properties, diverse shapes, sizes, and functionality, which can be precisely tailored by adjusting the plant source, extraction conditions, and synthesis process parameters [11, 12]. Moreover, green synthesis using plant-based materials is associated with reduced cytotoxicity and improved biocompatibility of the resulting AgNPs, as demonstrated by different authors, making them ideal candidates for antimicrobial applications [13, 14].

Pomegranate *(Punica granatum)*, a fruit extensively cultivated in the Mediterranean region, has long been employed for its therapeutic properties, including its antioxidant, antibacterial, and antitumor potential [15, 16]. These activities are primarily attributed to the presence of bioactive tannins. Notably, the ellagitannin punicalagin and its derivative ellagic acid, prevalent in pomegranate peel, are key antioxidant constituents, boasting remarkable free radical–scavenging capabilities [17]. These compounds, in conjunction with several other polyphenols, proteins, or sugars found in pomegranate peels, offer an exceptional blend of reducing and stabilizing agents, ideal for the green synthesis of various MNPs, including AgNPs [18–20], AuNPs [19], or ZnNPs [21] and metal oxide NPs such as ZnONPs [22, 23].

Optimization of MNP synthesis is a critical step in achieving desired features. However, it is both resource-intensive and time-consuming, requiring a substantial number of trials and the detailed overlooking of potential interactions between the process factors [24]. These limitations can be more effectively addressed by employing a multilevel statistical design, such as the response surface methodology (RSM). RSM not only reduces the overall number of experiments but also facilitates the creation of appropriate models for process optimization, resulting in a more comprehensive understanding of the interactions between the variables [25]. Furthermore, RSM quantifies the relationship between the controllable input parameters and the resulting response surfaces [26]. One notable type of RSM is the Box–Behnken design (BBD), which is renowned for its effectiveness in establishing correlations between response outcomes and the relevant factors through a carefully structured sequence of experiments, ultimately leading to the identification of the most favorable responses [27].

The present study focuses on the optimization of the green synthesis of AgNPs using *P. granatum* extract (PGE) as the reducing and stabilizing agent. A BBD analysis for three factors is performed using a Jupyter Notebook hosted by Colab programmed in Python language created specifically for the work. It employs BBD to systematically investigate and fine-tune the process variables, including temperature, AgNO_3_ concentration, and PGE concentration, to enhance the quality and reproducibility of AgNPs production. The optimized AgNPs were thoroughly characterized, and their antimicrobial activity was evaluated. Finally, as a proof of concept, the AgNPs were incorporated into nanofibers (NFs), demonstrating their potential as a platform for targeted delivery.

## 2. Experimental Section

### 2.1. Reagents and Materials

Silver nitrate (AgNO_3_), aluminum chloride (AlCl_3_), Folin–Ciocalteu's phenol reagent, 2, 2′-azinobis (3-ethylbenzothiazolin)-6-sulfonic acid (ABTS), gallic acid, glucose, 6-hydroxy-2,5,7,8-tetramethylchroman-2-carboxylic acid (Trolox), potassium bromide (KBr), potassium persulfate (K_2_S_2_O_8_), Coomassie Brilliant Blue G-250, 3,5-dinitrosalicylic acid (DNS), sodium potassium tartrate, phenol, sodium metabisulfite, and p-iodonitrotetrazolium chloride (INT) were purchased from Sigma-Aldrich (St. Louis, MO, USA). Punicalagin molecular standard was supplied by Merck (Darmstadt, Germany). Müller–Hinton (MH) broth was provided by Condalab (Madrid, Spain). As in a previous study by our group [28], the bacterial species *Escherichia coli* (CECT 515) and *Staphylococcus aureus* (CECT 59), obtained from the Spanish Type Culture Collection (CECT, Valencia, Spain), were used.

### 2.2. Extract Preparation and Characterization

#### 2.2.1. Preparation of PGE

The PGE employed in this study was prepared in the laboratory from organic pomegranate peel of the “Mollar de Elche” variety collected in Elche (Alicante, Spain), which holds Protected Designation of Origin (PDO) status. First, the peels were washed, dried at 60°C in an ZHWY-100B incubator oven (Zhicheng Instruments, Shanghai, China), and grounded using a MF10 basic mill (IKA, Staufen, Germany) to obtain uniform particle sizes of 1–2 mm. Afterward, an ultrasound-assisted extraction was performed by providing an energy of 100 J/mL to the extraction medium, which consisted of distilled water at 60°C, using a UP400 St ultrasonicator (Hielscher, Teltow, Germany). After one hour of agitation, the extract was filtered using a Colombo plate filter with V4 filters (Rover Pompe, Polverara, Italy), taken to a *R*-220 Pro rotary evaporator (Büchi, Flawil, Switzerland) to concentrate, and finally dried to powder form using a *B*-290 spray-dryer (Büchi).

#### 2.2.2. Phenolic Content Determination

The total phenolic content of the extracts was measured by using the Folin–Ciocalteu method in 96-well plates as previously described [29]. Briefly, 10 μL of each sample was mixed with 50 μL of Folin–Ciocalteu's phenol reagent. After 1 min, 100 μL of Na_2_CO_3_ solution (20%, *w*/*v*) and 840 μL of distilled water were added to the mix. The reaction was kept in dark for 30 min. Plate absorbance was measured at 700 nm using a BioTek Synergy HTX Multimode Microplate Reader (Agilent Technologies, Santa Clara, CA, USA). A standard curve of gallic acid was used for calibration, and results were expressed as gallic acid equivalents (g GAE/100 g of dry extract).

#### 2.2.3. Total Flavonoids Quantification

The quantification of flavonoids was performed using the aluminum chloride (AlCl_3_) colorimetric method [30], which involves the formation of a flavonoid–aluminum complex producing a yellow color measurable by spectrophotometry. Briefly, 0.5 mL of PGE was mixed with 4.5 mL of 0.2% AlCl_3_ methanolic solution containing 0.1 mL of potassium acetate 1 M and allowed to react for 30 min at room temperature. The absorbance of the resulting solution was measured at 415 nm using a BioTek Synergy HTX Multimode Microplate Reader. A standard curve was generated using quercetin as a reference, and the flavonoid content in the samples was expressed as g of quercetin equivalents per 100 g of dry extract.

#### 2.2.4. High-Performance Liquid Chromatography (HPLC)

The molecular composition of the PGE was analyzed by HPLC using an Agilent LC 1100 series (Agilent Technologies). Briefly, HPLC instrument was equipped with a pump, autosampler, UV-vis diode array detector (DAD), and column oven. The HPLC instrument was controlled by Chemstation software. The chromatographic column used was an Agilent Poroshell 120 RP-C18 column (4.6 × 150 mm, 2.7 μm). The method used for PGE components separation consisted of a linear gradient of 1% formic acid (A) and acetonitrile (B). Gradient started at 5% of B, increasing to 25% of B at 30 min, to 45% of B at 45 min, then 5% of B at 51 min and for an additional 5 min for column re-equilibration purposes. The flow rate was constant at 0.5 mL/min. The diode-array detector was set at 280, 320, and 340 nm.

Identification of the main compounds was performed by HPLC-DAD analysis using a home-made library of phenolic compounds and comparing the retention times and UV spectra data of the peaks in the samples with those of authentic standards or data reported in the literature. Punicalagin molecular standard was used to identify the main component of PGE. The interpretation of the spectra and identification of the main compounds was carried out using OpenChrom 1.4 software (Lablicate GmbH, Hamburg, Germany).

#### 2.2.5. Antioxidant Determination

The antioxidant capacity of the PGE was determined by the Trolox Equivalent Antioxidant Capacity (TEAC) assay. In this method, the radical precursor ABTS, pretreated with potassium persulfate, produced the radical cation (ABTS^•+^) after 12–24 h of incubation at room temperature. For the study of extract, the ABTS^•+^ solution was diluted with water to an absorbance of 0.70 (±0.02) at 734 nm (approximately 45 μM). ABTS^•+^ underwent a reduction process proportional to the antioxidant capacity of each extract sample. This reaction involves a loss of color intensity that was quantified at a wavelength of 734 nm. A calibration curve was prepared with different concentrations of Trolox up to 10 μM. Results are presented in mmol of Trolox equivalents per 100 g of dry extract [29].

#### 2.2.6. Protein Quantification

The protein content of PGE was quantified using the method described by Bradford [31] with some modifications. The method is based on the binding of Coomassie Brilliant Blue G-250 dye to proteins, which results in a shift of the dye's absorbance maximum from 465 nm to 595 nm. A standard curve was generated using known concentrations of bovine serum albumin (BSA) to determine the protein concentration in the samples. A volume of 200 μL of Coomassie Brilliant Blue G-250 was added to each standard curve or sample tube to achieve a total volume of 1 mL. Absorbance readings at 595 nm were measured using a BioTek Synergy HTX Multimode Microplate Reader.

#### 2.2.7. Total Reducing Sugars' Quantification

The quantification of reducing sugars was carried out using the DNS method described by Miller [32] with some modifications. This colorimetric assay relies on the reduction of DNS by free aldehyde or ketone groups present in reducing sugars, resulting in the formation of 3-amino-5-nitrosalicylic acid, which exhibits a reddish-brown color. DNS reagent was prepared by dissolving 1.87 g of 3, 5-dinitrosalicylic acid and 3.48 g of NaOH in distilled water. To this solution, 53.9 g of sodium potassium tartrate, 1.34 mL of phenol, and 1.46 g of sodium metabisulfite were added. The final volume was adjusted to 250 mL with distilled water. A standard curve was established using known concentrations of glucose as a reference. In the assay, 1 mL of the standard or PGE samples was mixed with 1 mL of DNS reagent and incubated in a boiling water bath for 5 min to allow the reaction to proceed. After cooling to room temperature, the absorbance of the resulting solution was measured at 540 nm using a BioTek Synergy HTX Multimode Microplate Reader.

### 2.3. AgNPs Synthesis

A 0.1M AgNO_3_ stock solution and a 50mg/mL pomegranate extract stock solution were initially prepared in Milli-*Q* water. To synthesize the AgNPs, different volumes of the PGE (ranging from 0.16 to 0.48 mL, equivalent to 0.16–0.48 mg/mL in the final reaction) were added into solutions of 5–15 mM AgNO_3_, which were derived from the initial stock solution. The total volume of the reaction mixtures consistently remained at 50 mL. The solution was stirred with the help of a magnetic stirrer, with temperatures maintained within the range of 20°C–80°C, depending on the experiment, for 24 h. Following this, the AgNPs were subjected to centrifugation at 15,000 rpm for 30 min. The supernatant was discarded, and precipitated AgNPs were redissolved in Milli-Q water and sonicated for 15 min. This process was repeated twice. The resulting AgNPs were subjected to freeze-drying to facilitate subsequent characterization requiring solid material.

### 2.4. RSM

A three-factor, three-level BBD was employed to optimize AgNPs using a Jupyter Notebook hosted by Colab programmed in Python language created specifically for the work. The program uses Python Design of Experiment Generator in Python (DOEPY) library. The BBD comprised 15 experimental trials, incorporating three replicated central points. The independent variables consisted of AgNO_3_ concentration (*X*_1_), PG extract concentration (*X*_2_), and reaction temperature (*X*_3_). These three variables were manipulated at three different levels: low, medium, and high. The upper and lower limits for the experimental factors were determined based on the existing literature and preliminary experiments. The responses or dependent variables under investigation encompassed hydrodynamic diameter (HDD) (*Y*_1_), polydispersity index (PDI) (*Y*_2_), and zeta potential (ZP) (*Y*_3_). The optimization of AgNPs synthesis was based on achieving the minimum possible values for each of the responses. The variations in the process parameters are summarized in Supporting Table S1.

### 2.5. Nanoparticle Characterization

#### 2.5.1. Microscopy

A single drop (approximately 10 μL) of the different AgNPs solutions was dispensed onto a silicon wafer and allowed to dry. The morphological structure of the optimal synthesized AgNPs were analyzed by field emission scanning electron microscopy (FESEM) with a Schottky hot cathode field emission model Sigma 300 VP apparatus (Carl Zeiss Microscopy GmbH, Oberkochen, Germany) with a coupled energy dispersive X-ray system (EDX) to determine their element composition. Samples were imaged under 1 or 20 kV electron high tension (EHT) and using a secondary electron detector (SE2) or a backscattered electron detector (AsB), depending on the sample.

#### 2.5.2. UV-Vis Spectrophotometry

The UV-Vis spectrum of the synthesized aqueous AgNPs was acquired using a 1-cm path length quartz cell on a Shimadzu UV-1700 (Kyoto, Japan) UV-Vis spectrophotometer. The absorbance of the solutions was measured at room temperature in the 250–800-nm range with a resolution of 1 nm.

#### 2.5.3. Dynamic Light Scattering (DLS)

Particle size and surface charge analyzer Zetasizer Nano-ZS ZEN 3600 (Malvern Instruments Limited, Worcestershire, United Kingdom) was used to determine the HDD and PDI through DLS. AgNPs samples were placed into *U*-shaped capillary cells for ZP analysis. The analysis was done at 25°C in triplicate. Prior to the analysis, the AgNPs were suspended in Milli-*Q* water and sonicated for 10 min.

#### 2.5.4. Fourier Transform Infrared (FTIR) Spectroscopy

The chemical composition of the samples was assessed through FTIR spectroscopy using a Spectrum Two FTIR spectrometer (PerkinElmer, Waltham, MA, USA). Two milligrams of the dried samples were pulverized with KBr salt at 25°C and compressed into a mold to create a pellet. The spectra were captured within a wavelength range of 450–4000 cm^−1^ at a resolution of 4 cm^−1^.

#### 2.5.5. *X*-Ray Diffraction Analysis (XRD)

The XRD pattern was collected on a D8 Advance diffractometer (Bruker, Karlsruhe, Germany) equipped with a 1D LynxEye detector, using Ni-filtered Cu Kα radiation. The powder sample was mounted in a low background off-cut silicon crystal sample holder. The XRD pattern was collected using Bragg–Brentano geometry, at room temperature, by scanning in the angle range 5° ≤ 2*θ* ≤ 80° with a step of 0.01° and a dwell of 1 s per step.

### 2.6. Antibacterial Assays

The antibacterial efficacy of AgNPs and AgNP-loaded NFs was assessed at the BSL-2 facilities of the General University Hospital of Alicante through the broth microdilution technique to determine the minimum inhibitory concentration (MIC), as in our previous work with some modifications [33]. Briefly, to determine the MIC, dried AgNPs were initially dissolved in Milli-*Q* water. Subsequently, 50 μL of this solution was added to the wells of a 96-well plate, with concentrations starting from 80 μg/mL and serially diluted by half. In addition, 20 μL of a bacterial suspension at 0.5 McFarland was added to each well, followed by MH broth to reach a final volume of 200 μL per well. The plates were then incubated at 37°C for 24 h. Following incubation, 50 μL of a 1mg/mL INT solution was introduced into each well and allowed to incubate for 30 min. The MIC was defined as the lowest concentration at which no red color was observed.

### 2.7. Observation of Bacterial Cell Morphology After AgNPs Exposure

Bacterial cells, both untreated and treated with the MIC for 24 h, were fixed on borosilicate cover glass slides according to the protocol described by Pulingam et al. Briefly, the cells were treated with 4% glutaraldehyde (GLA) for 30 min, washed with PBS, and further fixed with 1% osmium tetroxide for an additional 30 min. Samples were progressively dehydrated using ethanol (30%, 50%, 70%, 80%, 90%, and 100% for 15 min each). Micrographs of the bacteria were captured using a Sigma 300 VP FESEM (Carl Zeiss, Germany) at 1 kV without coating. EDX was employed to determine the presence of AgNPs inside the bacterial cells.

### 2.8. Nanoparticle Incorporation Into NFs

The optimized AgNPs (AgNPs-OPT) were selected to be incorporated into NFs as proof of concept, to evaluate their potential as scaffolds for biocidal applications. AgNPs (0.2% *w*/*w*) were added to a polyethylene oxide (PEO, 3% *w*/*w*) and BSA (15% *w*/*w*) solution in distilled water, as previously described [33]. The solutions were left stirring overnight. Finally, the NFs were synthesized via electrospinning. For that purpose, the solutions were loaded into 2 mL syringes (Becton Dickinson, Franklin Lakes, NJ, USA) connected to a blunt-end stainless steel needle (5 cm long, outer diameter of 1.27 mm and inner diameter of 0.84 mm, Sigma-Aldrich). Electrospinning was conducted with a controlled flow rate of 0.25 mL/h using a KDS 100 infusion pump (KD Scientific, Holliston, MA, USA), a voltage of 18 kV using high voltage source (Glassman High Voltage Inc., Whitehouse Station, NJ, USA), and a tip-to-collector distance of 17 cm, under conditions of 20%–40% relative humidity and 25°C. The obtained NFs were deposited in glass slides and characterized through FESEM after chrome coating (Q150T S plus, Quorum Tech., United Kingdom). The average diameter of the NFs was determined using Image *J* (National Institutes of Health, Bethesda, MD, USA) image processing and analysis software from 100 measurements using different images.

### 2.9. Data Analysis

All experimental data from BBD were collected in triplicate and expressed as mean values ± standard deviation (S.D.). Graphs were carried out using Prism v8 software (GraphPad software, La Jolla, CA, USA), unless stated otherwise.

## 3. Results and Discussion

### 3.1. PGE Characterization

The total polyphenolic content of PGE, as determined by the Folin–Ciocalteu method, showed a value of 29.94 ± 0.36 g of GAE per 100 g of dry extract. This significant concentration indicates a robust presence of phenolic compounds, which are known for their antioxidant properties. Comparatively, green coffee extract typically exhibits polyphenolic content around 25–31 g GAE/100 g dry weight [34], and olive oil shows approximately 31–39 g GAE/100 g dry weight [35]. These values underscore the substantial polyphenolic concentration found in pomegranate peel when compared with other known polyphenolic-rich matrices.

**Figure 1:**
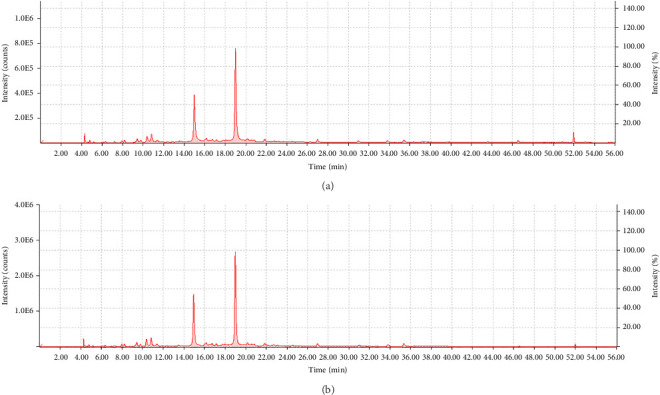
PGE chromatogram obtained by HPLC. PGE samples were analyzed at a concentration of (a) 1 mg/mL and (b) 3 mg/mL.

To accurately determine the polyphenolic profile of PGE, different HPLC analyses were performed. The chromatograms of PGE at concentrations of 1 mg/mL and 3 mg/mL can be observed in Figures 1(a) and 1(b), respectively.

To identify and quantify the main components of the PGE chromatogram, a punicalagin molecular standard was used. This standard was chosen based on the previous experience of the group and bibliography. The chromatograms of the punicalagin molecular standard analyzed at different concentrations, as well as the regression line (*R*^2^ = 0.9961) calculated based on the areas of the peaks obtained, can be observed in Supporting Figures S1 and S2, respectively.

The analysis of the retention times and DAD of the molecular standard demonstrated that the main polyphenol present in the PGE is punicalagin, representing 29.45 ± 1.76% of the total dry weight of the extract. This value means that punicalagin accounts for most of the total polyphenols measured by the Folin–Ciocalteu assay, which is consistent with the chromatograms obtained, in which the peaks corresponding to punicalagin are those that show greater intensity and area. These results agree with previous studies on pomegranate peel extracts, in which hydrolyzable tannins quantified as punicalagin equivalents by HPLC represented 30.46% of the total dry weight of the extract [16]. The flavonoid content of PGE was determined, revealing a total of 5.15 ± 0.33 g of quercetin equivalents per 100 g of dry extract. This value is consistent with the chromatographic profile obtained from PGE, in which the major peaks belong to hydrolyzable tannins such as punicalagin, and the minor peaks could be related to flavonoids.

The PGE used in this work exhibits a remarkably high antioxidant capacity, quantified at 1379 ± 40 mmoles TROLOX equivalents per 100 g of dry extract. This value is notably higher when compared to other plant extracts. For instance, the antioxidant capacity of green tea extract, a well-known antioxidant source, is around 460 mmoles TROLOX equivalents per 100 g, and other extracts of popular herbs such as the ones from *Ilex paraguariensis* (mate) or *Salvia rosmarinus* (rosemary) range from 128 to 154 mmoles TROLOX equivalents per 100 g [36]. The high antioxidant capacity of PGE can be attributed to the presence of highly active phenolic compounds such as punicalagins [37]. The analysis of total reducing sugars in PGE showed a concentration of 57.2 g glucose equivalents per 100 g of dry extract. This finding is consistent with the overall composition of pomegranate peel, which is known to be rich in various sugars [38]. The total protein concentration in PGE was 0.84 ± 0.07 g BSA equivalents per 100 g of dry extract, accounting for 0.84 ± 0.07% of the PGE. This value is lower than other pomegranate peel extracts, which report values between 3.08 ± 0.02% [39] and 4.98 ± 0.50% [38]. These differences can be attributed to differences between pomegranate varieties and extraction methods.

### 3.2. BBD

#### 3.2.1. Response Surface Plot

**Figure 2:**
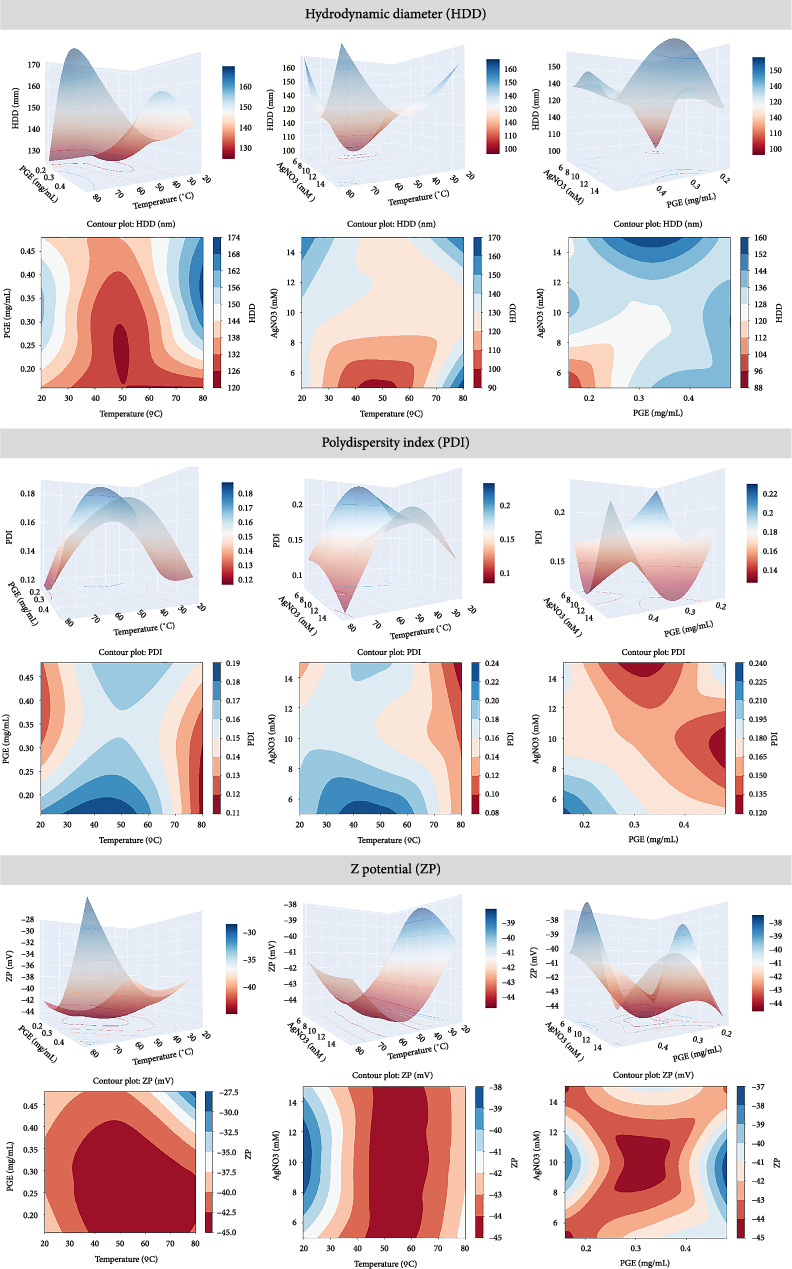
3D response surface plots and 2D contour plots of Box–Behnken statistical experimental design showing the interaction effects of temperature, PGE concentration, and AgNO_3_ concentration on HDD, PDI, and ZP as response variables.

Response surface analysis was conducted employing 3D response surface plots and 2D contour plots to elucidate the contribution of independent variables to various response variables.  Figure 2 illustrates the response surface analysis plots for HDD, PDI, and ZP.

Regarding HDD, it can be observed that at moderate temperatures (around 50°C), the parameter is minimized in both temperature-PGE and temperature-AgNO_3_ plots. The effect of PGE and AgNO_3_ concentrations shows an increase in HDD with the rise in concentration in both cases.

For PDI, the relationship between temperature and PGE exhibits a curvilinear trend, showcasing minimal levels at lower temperatures alongside moderate to high PGE concentrations, followed by a decrease and subsequent increase at higher temperatures and moderate to low PGE concentrations. The relationship between temperature and AgNO_3_ indicates that PDI is minimized at high temperatures and high concentrations of AgNO_3_. Finally, the correlation between PGE and AgNO_3_ unveils two separate minimum points: one occurring at moderate PGE concentrations and high AgNO_3_ concentrations, and the other at high PGE concentrations and moderate AgNO_3_ concentrations.

As for ZP, it appears to be the most minimized response in all cases. The relationship between temperature and PGE shows minimal ZP values under almost all conditions, although it is favored at medium to high temperature values and medium to low concentrations of PGE. The temperature–AgNO_3_ relationship demonstrates that at intermediate temperature values (50°C–60°C), ZP is minimized without influence from AgNO_3_ concentration. Finally, the plot of PGE and AgNO_3_ concentrations displays minimal ZP values at intermediate concentrations in both cases.

#### 3.2.2. Polynomial Model for Each Response

The obtained data were used to define polynomial models for each response (*Y*_1_, HDD; *Y*_2_, PDI; *Y*_3_, ZP), which demonstrate interaction among all the analyzed variables and are described in Supporting Table S2.

**Figure 3:**
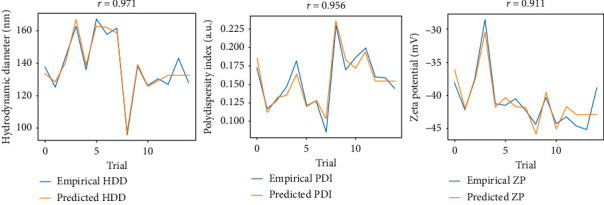
Comparison between the results predicted by the model (orange line) and the empirical results obtained in the 15 trials (blue line). *r* corresponds to Pearson's correlation coefficient.

Furthermore, a prediction–response comparison was performed, as shown in Figure 3. All parameters exhibited a Pearson correlation coefficient (*r*) greater than 0.9, with values of 0.971, 0.956, and 0.911 for HDD, PDI, and ZP, respectively. These values indicate a strong association among the variables, thus indicating a good fit of the data to the selected model [40].

#### 3.2.3. Optimal AgNPs Production Conditions

The study aimed to identify optimal conditions that would minimize all investigated responses simultaneously. A particular focus was placed on minimizing the HDD of the AgNPs. Smaller NPs possess a larger surface area to volume ratio, which facilitates increased interaction with bacteria [41], thereby enhancing their antibacterial properties. In addition, minimization of PDI was also pursued since values below 0.2 generally indicate a monodisperse distribution, which is desirable for consistent material properties [42]. Finally, a ZP below −30.0 mV was sought as it signifies stable AgNP dispersions [43]. These optimized NPs were designated as AgNPs-OPT.

In addition, conditions that optimized each parameter individually were also investigated. These include the smallest possible HDD (AgNPs-HDD), the lowest PDI (AgNPs-PDI), and the most negative ZP (AgNPs-ZP). Supporting Table S3 summarizes the conditions required to achieve optimal results in each case.

Subsequently, AgNPs were synthesized using the predicted conditions to validate the obtained results. The root-mean-square error (RMSE) was also calculated to allow evaluation of the predictive accuracy of the employed optimization model compared to other developed models in the future. These results are presented in Table 1. The graphical representations of the obtained experimental values can be found in Supporting Figure S3. As observed, the experimental values closely align with the model's predictions, demonstrating a strong fit of the selected design.

### 3.3. Nanoparticle Characterization

#### 3.3.1. Microscopy

**Figure 4:**
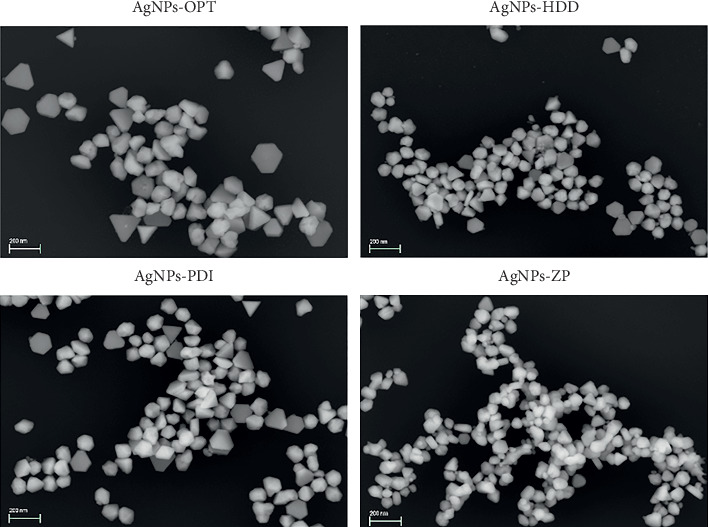
FESEM images (SE2 detector, EHT = 1 kV) of the AgNPS-OPT, AgNPs-HDD, AgNPs-PDI, and AgNPs-ZP. Scale bar: 200 nm.

The morphology of the synthesized AgNPs was characterized using FESEM, as represented in Figure 4. The images confirmed that the synthesized NPs were within the nanoscale range and exhibited mixed anisotropic morphologies, including spherical, quasi-spherical, triangular, and hexagonal shapes. This observation aligns with the literature, as green-synthesized AgNPs are known to exhibit diverse shapes and sizes, with the most common forms being spherical, triangular, and hexagonal [44]. Furthermore, EDX analysis confirmed the presence of silver associated to each AgNP in all samples (Supporting Figure S4).

#### 3.3.2. UV-Vis Spectrophotometry

**Figure 5:**
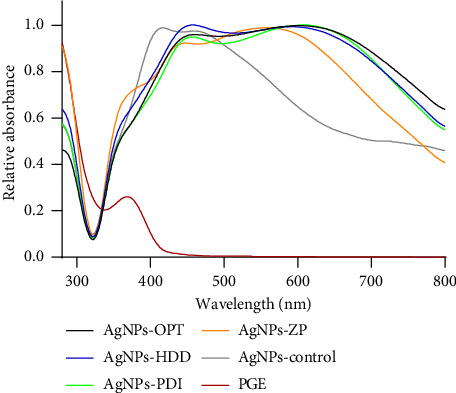
UV-vis spectrum of the four types of optimized green synthesized AgNPs and *Punica granatum* extract (PGE).

UV-Vis spectrophotometry was employed to investigate the absorption spectrum of the optimized green-synthesized AgNPs, as it provides information about the correct formation of the NPs. Spherical AgNPs typically exhibit a surface plasmon resonance (SPR) band extending in the range from 380 to 460 nm [45]. In the case of the AgNPs synthesized and optimized in this study (Figure 5), the maximum of the SPR band was observed in the range of 435–455 nm, closely resembling the commercial AgNPs spectrum (AgNPs-control), which displays two distinct peaks at 415 and 462 nm. Their absorption spectrum is associated with the typical spectrum of NPs with a diameter close to 100 nm, as larger spheres tend to show increased scattering, leading to broadening and shifting of peaks toward longer wavelengths, a phenomenon known as red-shifting [46, 47]. Furthermore, the AgNPs synthesized via green synthesis using PGE exhibit more than one SPR band, indicating their anisotropic nature [48].

#### 3.3.3. FTIR

**Figure 6:**
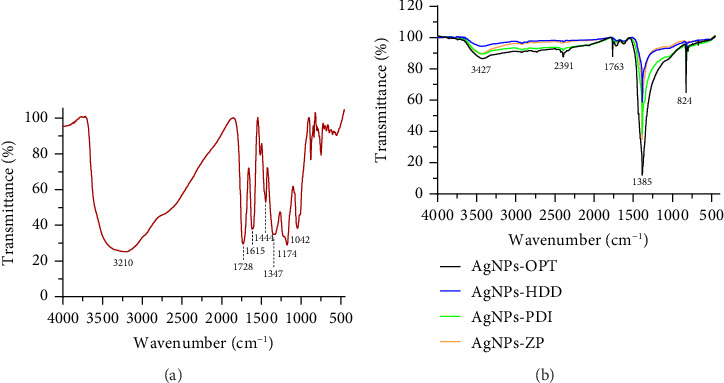
FTIR spectra of (a) *Punica granatum* extract (PGE) and (b) four types of optimized green synthesized AgNPs.

The FTIR analyses were conducted to identify the functional groups of the potential biomolecules involved in the capping and stabilization of the AgNPs synthesized with PGE. The FTIR spectra of PGE and the optimized AgNPs were recorded in the wavenumber range of 4000–450 cm^−1^ and are shown in Figures 6(a) and 6(b), respectively. The broad band observed in the FTIR spectrum of PGE (Figure 6(a)) at 3210 cm^−1^ is attributed to the characteristic O-H stretching vibration of phenolic groups [49]. Considering the high concentration of punicalagin in the utilized PGE, it is probable that the hydroxyl groups of this biomolecule predominantly account for the emergence of this spectral band. Other significant peaks were observed at 1728 cm^−1^ (C = O stretching) [50], 1615 cm^−1^ (C = C bending) [51], 1444 cm^−1^ (CH_2_ bending) [52], 1347, 1174, and 1042 cm^−1^ (C-O stretching) [53].

In the FTIR spectra of the optimized AgNPs, a prominent peak at 1385 cm^−1^ is consistently observed across all samples. This band is associated with commercial AgNO_3_, indicating potential traces of unreacted starting material, as well as the C-O stretching from PGE. In addition, the spectra of the optimized green-synthesized AgNPs display peaks similar to those of PGE but with slight shifts or variations in intensity, indicating interactions between the AgNPs and functional groups of the capping agents [54]. Notably, the peak at 3210 cm^−1^ shifts to 3427 cm^−1^, reflecting the bioreduction and stabilization of the AgNPs [55, 56]. The reduced intensity of the peak at 3210 cm^−1^ further highlights the crucial role of O-H and N-H groups in the reduction and binding mechanisms [54].

#### 3.3.4. XRD

**Figure 7:**
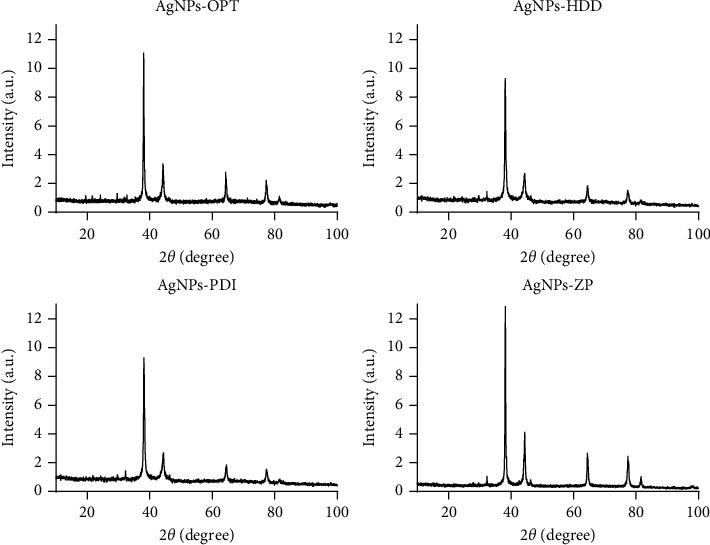
XRD pattern of the four types of optimized green synthesized AgNPs.

XRD revealed the crystalline nature of the different optimized AgNPs, as depicted in Figure 7. Diffracted intensities were recorded from 10° to 100°. Four intense Bragg reflections were identified around 38°, 44°, 64.5°, and 77.4°. These are associated with the (1 1 1), (2 0 0), (2 2 0), and (3 1 1) crystal planes, respectively, and their indexing can be performed according to the faces present in the face-centered cubic crystal structure of silver [57]. The presence of additional smaller peaks is attributed to the presence of AgNO_3_, the salt used in the synthesis of the NPs, which possibly remained as a residue in the optimized green-synthesized AgNPs [58].

### 3.4. Antibacterial Activity

AgNPs exhibit broad antimicrobial activity, and their potent activity is associated with their morphological and physicochemical characteristics such as size, shape, surface-to-volume ratio, surface charge, or crystallinity, among others [59]. In this study, two bacteria models of biomedical interest were selected to evaluate antibacterial activity of AgNPs: *E. coli* (Gram-negative bacteria) and *S. aureus* (Gram-positive bacteria).

Firstly, the MIC of the four types of optimized green-synthesized AgNPs was evaluated. The results obtained can be seen in Table 2. The MICs were similar among the different types of AgNPs and ranged from 2.5 to 10 μg/mL. These results stand out with respect to those described in the literature, where some authors have reported the MIC of AgNPs against *E. coli* in the range of 7.8–125 μg/mL [60–62] and that of *S. aureus* between 2–250 μg/mL [60, 62–64]. The results obtained place the optimized AgNPs obtained here among those with greater antibacterial activity against *E. coli* and *S. aureus*.

**Figure 8:**
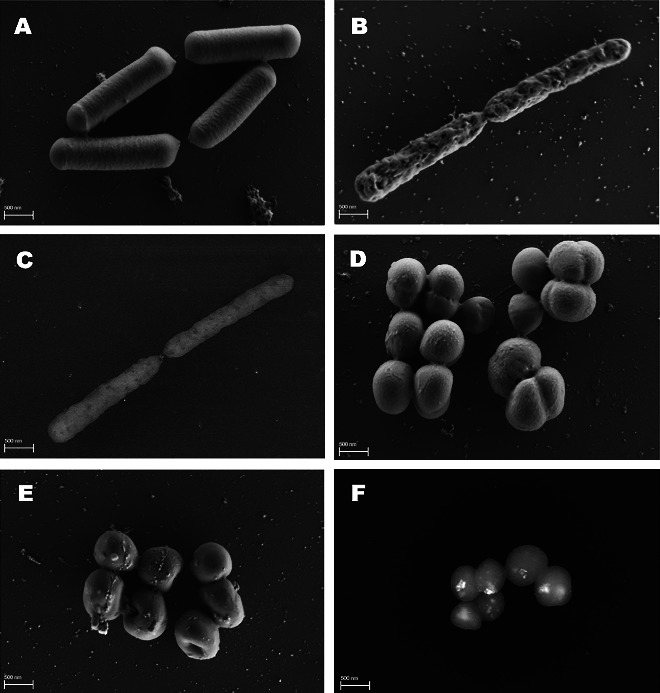
FESEM images showing (A) nontreated *E. coli* cells (SE2 detector, EHT = 1 kV), (B) *E. coli* cells treated with AgNPs-OPT at MIC (SE2 detector, EHT = 1 kV), (C) *E. coli* cells treated with AgNPs-OPT at MIC (AsB detector, EHT = 20 kV), (D) nontreated *S. aureus* cells (SE2 detector, EHT = 1 kV), (E) *S. aureus* cells treated with AgNPs-OPT at MIC (SE2 detector, EHT = 1 kV), and (F) *S. aureus* cells treated with AgNPs-OPT at MIC (AsB detector, EHT = 20 kV).

Subsequently, the effect of AgNPs-OPT on bacterial cell morphology was evaluated using FESEM. The results are presented in Figure 8, where Figures 8(A) and 8(B) show untreated and treated *E. coli*, respectively, and Figures 8(D) and 8(E) show untreated and treated *S. aureus*, respectively. The untreated bacteria were observed to have an intact cell membrane compared to bacterial cells treated with AgNPs. The bacteria treated with AgNPs exhibited irregularities in their morphology, depending on the type of bacteria tested. Gram-negative bacteria *E. coli* exhibited hollows and a more wrinkled surface compared to the control bacteria. Similar observations were reported by different authors, who found that cell membrane disruption occurred in *E. coli* following exposure to AgNPs [65–67], causing an increase in permeability and ultimately leading to bacterial cell death. In the present study, AgNPs appear to affect the cell membrane in *E. coli*, finally causing their death. However, no AgNPs were detected inside the Gram-negative bacterial cells after 24 h of exposure (Figure 8(C)). On the other hand, the Gram-positive bacteria *S. aureus* also exhibited morphological alterations, though membrane damage was not as significant as in *E. coli*. Vesicles appeared on the surface, which may suggest a small leakage of cytoplasmic components. In addition, invaginations were observed on the surface of *S. aureus*, likely due to the penetration of AgNPs into the bacterial cell (Figure 8(F)), as confirmed by EDX analysis in Supporting Figure S5. These findings are consistent with other studies that observed the presence of AgNPs within the cytoplasm of *S. aureus* [68] or other Gram-positive bacteria [69]. Once inside the cells, silver is known to deactivate respiratory enzymes, generate reactive oxygen species, and disrupt the production of adenosine triphosphate (ATP) [70]. However, more research is needed to fully understand the intracellular mechanisms of AgNPs.

### 3.5. AgNP Incorporation Into NFs

The incorporation of AgNPs into nanofibrous scaffolds presents a promising approach for their application in biomedical applications, particularly in combating bacterial infections [71]. In the present study, AgNPs-OPT were incorporated at 0.2% into solutions containing 15% BSA and 3% PEO for the synthesis of NFs by electrospinning. BSA was used in polymer solution because certain studies suggest that this protein can provide spatial stability for AgNPs and thus can be utilized as an effective protective agent to minimize their agglomeration [72].

**Figure 9:**
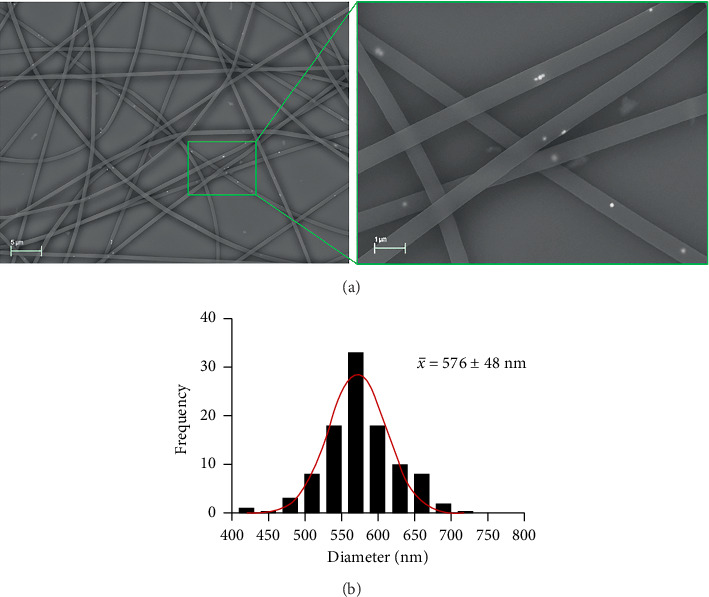
(a) FESEM images (AsB detector, EHT = 20 kV) of NFs synthesized from 3% PEO/15% BSA/0.2% AgNPs-OPT. AgNPs appear as white bright dots. Scale bar: 5 μm. Close up scale bar: 1 μm. (b) Diameter frequency histograms of electrospun NFs. Best-fit adjustments to a Gaussian distribution are indicated in red. A close-up image of the NFs is indicated in green (scale bar: 1 µm).

The NFs obtained are depicted in Figure 9. The resulting NFs exhibited an average diameter of 576 ± 48 nm and demonstrated uniformity in both morphology and size, as illustrated in the diameter frequency histogram in Figure 9(b). The appearance and morphology of the NF surface can be observed in Supporting Figure S6. The presence of AgNPs within the NFs is shown in Figure 9(a), which were randomly distributed within the NFs. The silver composition of the AgNPs within the NFs was confirmed with EDX analysis (Supporting Figure S7). In addition, some aggregation of AgNPs was observed in certain areas, which is common in one-step electrospinning processes of AgNPs with polymeric solutions [73].

Finally, the MIC of the NFs incorporating AgNPs-OPT was determined to be 10 μg/mL for both strains, based on the proportion of AgNPs within the NFs. Compared to the MIC of free AgNPs-OPT (Table 2), this represents an increase, suggesting that the antimicrobial activity was partially retained. This increase may be attributed to the low concentration of AgNPs within the NFs, leading to a nonhomogeneous distribution, as observed in the FESEM images. Future work will focus on optimizing the concentration of AgNPs to achieve the most effective antimicrobial platform.

## 4. Conclusion

In this study, AgNPs were synthesized using a green synthesis approach and optimized using a BBD created specifically for the work. This optimization strategy resulted in the development of four distinct types of AgNPs, each tailored to optimize either HDD, PDI, ZP, or all three parameters simultaneously. The characterization of the AgNPs confirmed the correct synthesis and a good fit of the BBD. The AgNPs displayed effective and differential antibacterial activity against *E. coli*, primarily targeting the bacterial membrane, and *S. aureus*, where the AgNPs were observed to penetrate the bacterial cells. As a proof of concept for potential antibacterial applications, the optimized AgNPs were incorporated into NFs, partially retaining their antimicrobial activity after incorporation. These results underscore the potential of BBD to engineer AgNPs with specific, desired characteristics, facilitating the development of novel antibacterial materials. Future research will focus on investigating how AgNPs characteristics affect their mechanism of action, refining the concentration of AgNPs into synthesized NFs, and further adjusting other critical parameters to enhance their effectiveness in antimicrobial applications.

## Figures and Tables

**Table 1:** Predicted and experimentally obtained values, as well as the RMSE for each of the responses related to the optimized conditions.

	Response	Predicted value	Experimental value	RMSE
AgNPs-OPT	HDD (nm)	131.3	130.3 ± 12.0	9.9
	PDI	0.147	0.149 ± 0.018	0.015
	ZP (mV)	−46.0	−48.3 ± 0.6	2.3

AgNPs-HDD	HDD (nm)	96.7	96.4 ± 0.5	0.5

AgNPs-PDI	PDI	0.092	0.115 ± 0.006	0.024

AgNPs-ZP	ZP (mV)	−46.3	−44.6 ± 1.8	2.2

**Table 2:** Antibacterial activity of four types of optimized green synthesized AgNPs, measured as minimum inhibitory concentration (μg/mL) against *E. coli* and *S. aureus*.

	AgNPs-OPT (μg/mL)	AgNPs-HDD (μg/mL)	AgNPs-PDI (μg/mL)	AgNPs-ZP (μg/mL)
*E. coli*	5.0	2.5	5.0	2.5
*S. aureus*	2.5	2.5	5.0	10

## Data Availability

The data that support the findings of this study are available from the corresponding author upon reasonable request.

## Supporting Information

Table S1: Experimental design layout of the BBD for the green synthesis of AgNPs using pomegranate extract (PGE). Table S2: Polynomial models for the responses (*Y*_1_: HDD; *Y*_2_: PDI; *Y*_3_: ZP) investigated in the BBD. Table S3: Conditions required to achieve optimal results that simultaneously minimize the three investigated responses (HDD, PDI, and ZP) (AgNPs-OPT), as well as individually (AgNPs-HDD, AgNPs-PDI, and AgNPs-ZP). Figure S1: Chromatogram of the molecular standard of punicalagin (A) at 0.1 mg/mL, (B) at 0.3 mg/mL, and (C) at 0.5 mg/mL. Figure S2: Graphical representation of the standard line of punicalagin used for the quantification of PGE. Figure S3: (A) Size distribution of AgNPs-OPT, (B) zeta potential analysis of AgNPs-OPT, (C) size distribution of AgNPs-HDD, (D) size distribution of AgNPs-PDI, and (E) zeta potential analysis of AgNPs-ZP. Figure S4: (A) FESEM image of AgNPs-OPT used for elemental mapping, (B) EDX analysis of AgNPs-OPT, (C) FESEM image of AgNPs-HDD used for elemental mapping, (D) EDX analysis of AgNPs-HDD, (E) FESEM image of AgNPs-PDI used for elemental mapping, (F) EDX analysis of AgNPs-PDI, (G) FESEM image of AgNPs-ZP used for elemental mapping, and (H) EDX analysis of AgNPs-ZP. Figure S5: (A) FESEM image of treated *S. aureus* used for elemental mapping and (B) EDX analysis of AgNPs found inside of *S. aureus*. Figure S6: FESEM image (SE2 detector, EHT = 1 kV) of the surface of NFs synthesized from 3% PEO/15% BSA/0.2% AgNPs-OPT. Figure S7: (A) FESEM image of NFs synthesized from 3% PEO/15% BSA/0.2% AgNPs-OPT used for elemental mapping and (B) EDX analysis of AgNPs found inside of the NFs.
