# Supplementary material for: An Innovative Approach Based on the Green Synthesis of Silver Nanoparticles Using Pomegranate Peel Extract for Antibacterial Purposes
Source: Bioinorg Chem Appl. 2025 Mar 13;2025:2009069. doi: 10.1155/bca/2009069 (PMC11925634; doi:10.1155/bca/2009069)
Supplement: Supporting Information — Additional supporting information can be found online in the Supporting Information section. [file 2009069.f1.docx]

**An innovative approach based on the green synthesis of silver nanoparticles using pomegranate peel extract for antibacterial purposes.**

**Rocío Díaz-Puertas^1^, Francisco J Álvarez-Martínez^1^, Enrique Rodríguez-Cañas^1^, Fernando Borrás^2^, Artur JM Valente^3^, José A Paixao^4^, Alberto Falcó^5^, Ricardo Mallavia^1*^**

1 Institute of Research, Development and Innovation in Healthcare Biotechnology in Elche (IDiBE), Miguel Hernández University, 03202 Elche, Alicante, Spain.

2 Statistics and Operative Research Department, Miguel Hernández University, 03202 Elche, Alicante, Spain.

3 Department of Chemistry, University of Coimbra, 3004-535 Coimbra, Portugal.

4 Department of Physics, University of Coimbra, 3004-516 Coimbra, Portugal.

5 Fish Pathology Group, Institute of Aquaculture Torre de la Sal — Spanish National Research

Council (IATS-CSIC), 12595 Cabanes, Castellón, Spain.

* Corresponding author: [r.mallavia@umh.es](mailto:r.mallavia@umh.es).

**Table S-1**. Experimental design layout of the Box-Behnken Design (BBD) for the green synthesis of AgNPs using pomegranate extract (PGE). The dependent variables are represented as mean ± S.D. of three measures. X_1_: temperature (^o^C); X_2_: PGE concentration (mg/mL); X_3_: AgNO_3_ concentration (mM); Y_1_: hydrodynamic diameter (HDD, nm); Y_2_: polydispersity index (PDI), Y_3_: zeta potential (ZP, mV).

| **Trial** | **Independent variables** | | | **Dependent variables** | | |
| --- | --- | --- | --- | --- | --- | --- |
|  | **X_1_** | **X_2_** | **X_3_** | **Y_1_** | **Y_2_** | **Y_3_** |
| 1 | 20 | 0.16 | 10 | 137.50±0.38 | 0.172±0.016 | -38.10±0.67 |
| 2 | 80 | 0.16 | 10 | 125.10±1.94 | 0.116±0.016 | -42.20±1.04 |
| 3 | 20 | 0.48 | 10 | 143.90±1.42 | 0.128±0.015 | -37.50±0.70 |
| 4 | 80 | 0.48 | 10 | 162.80±1.67 | 0.148±0.012 | -28.50±0.53 |
| 5 | 20 | 0.32 | 5 | 135.60±0.57 | 0.182±0.021 | -41.30±0.69 |
| 6 | 80 | 0.32 | 5 | 167.10±0.23 | 0.121±0.018 | -41.50±1.23 |
| 7 | 20 | 0.32 | 15 | 157.60±2.08 | 0.127±0.014 | -40.50±1.60 |
| 8 | 80 | 0.32 | 15 | 161.50±1.16 | 0.085±0.019 | -42.30±0.29 |
| 9 | 50 | 0.16 | 5 | 95.95±0.54 | 0.230±0.018 | -44.40±0.15 |
| 10 | 50 | 0.48 | 5 | 138.40±0.56 | 0.169±0.019 | -40.30±0.38 |
| 11 | 50 | 0.16 | 15 | 126.10±1.34 | 0.186±0.003 | -44.30±0.44 |
| 12 | 50 | 0.48 | 15 | 130.30±1.31 | 0.199±0.026 | -43.20±0.10 |
| 13 | 50 | 0.32 | 10 | 126.80±0.67 | 0.160±0.008 | -44.60±0.44 |
| 14 | 50 | 0.32 | 10 | 143.00±2.30 | 0.159±0.013 | -45.20±0.51 |
| 15 | 50 | 0.32 | 10 | 127.70±2.98 | 0.144±0.005 | -38.90±0.78 |

**Table S-2**. Polynomial models for the responses (Y_1_: HDD; Y_2_: PDI; Y_3_: ZP) investigated in the Box Behnken Design. The first term of the equations corresponds to the intercept, while the rest of the terms are made up of a coefficient that multiplies one or two of the variables (X_1_, temperature; X_2_, PGE; X_3_, AgNO_3_).

| **Response** | **Polynomial model** |
| --- | --- |
| **HDD** | Y_1_ = 76.204 + 5.761·X_1_ + 395.638·X_2_ - 2.253·X_3_ - 11.953·X_1_X_2_ - 0.046·X_1_X_3_ + 1.630·X_2_X_3_ + 0.066·X_1_^2^ - 447.998·X_2_^2^ + 0.024·X_3_^2^ |
| **PDI** | Y_2_ = 0.433 - 0.0234·X_1_ - 1.150·X_2_ + 0.002·X_3_ + 0.023·X_1_X_2_ + 3.416·10^-5^·X_1_X_3_ + 0.004·X_2_X_3_ + 0.001·X_1_^2^ + 1.0531·X_2_^2^-4.477·10^-5^·X_3_^2^ |
| **ZP** | Y_3_ = -30.619 + 2.353·X_1_ - 67.943·X_2_ - 0.623·X_3_ - 0.938·X_1_X_2_ - 0.003·X_1_X_3_ +  0.682·X_2_ X_3_ - 0.099·X_1_^2^ + 91.309·X_2_^2^ + 0.004·X_3_^2^ |

**Table S-3**. Conditions required to achieve optimal results that simultaneously minimize the three investigated responses (HDD, PDI, and ZP) (AgNPs-OPT), as well as individually (AgNPs-HDD, AgNPs-PDI, AgNPs-ZP). Grey slots inidcate non-measured parameters.

|  | **Temperature (^o^C)** | **PGE concentration (mg/mL)** | **AgNO_3_ concentration (mM)** | **Predicted value** | | |
| --- | --- | --- | --- | --- | --- | --- |
|  |  |  |  | **HDD (nm)** | **PDI** | **ZP (mV)** |
| AgNPs-OPT | 61 | 0.200 | 15.0 | 131.3 | 0.147 | -46.0 |
| AgNPs-HDD | 47 | 0.160 | 5.0 | 96.7 |  |  |
| AgNPs-PDI | 80 | 0.257 | 12.7 |  | 0.092 |  |
| AgNPs-ZP | 58 | 0.182 | 5.0 |  |  | -46.3 |


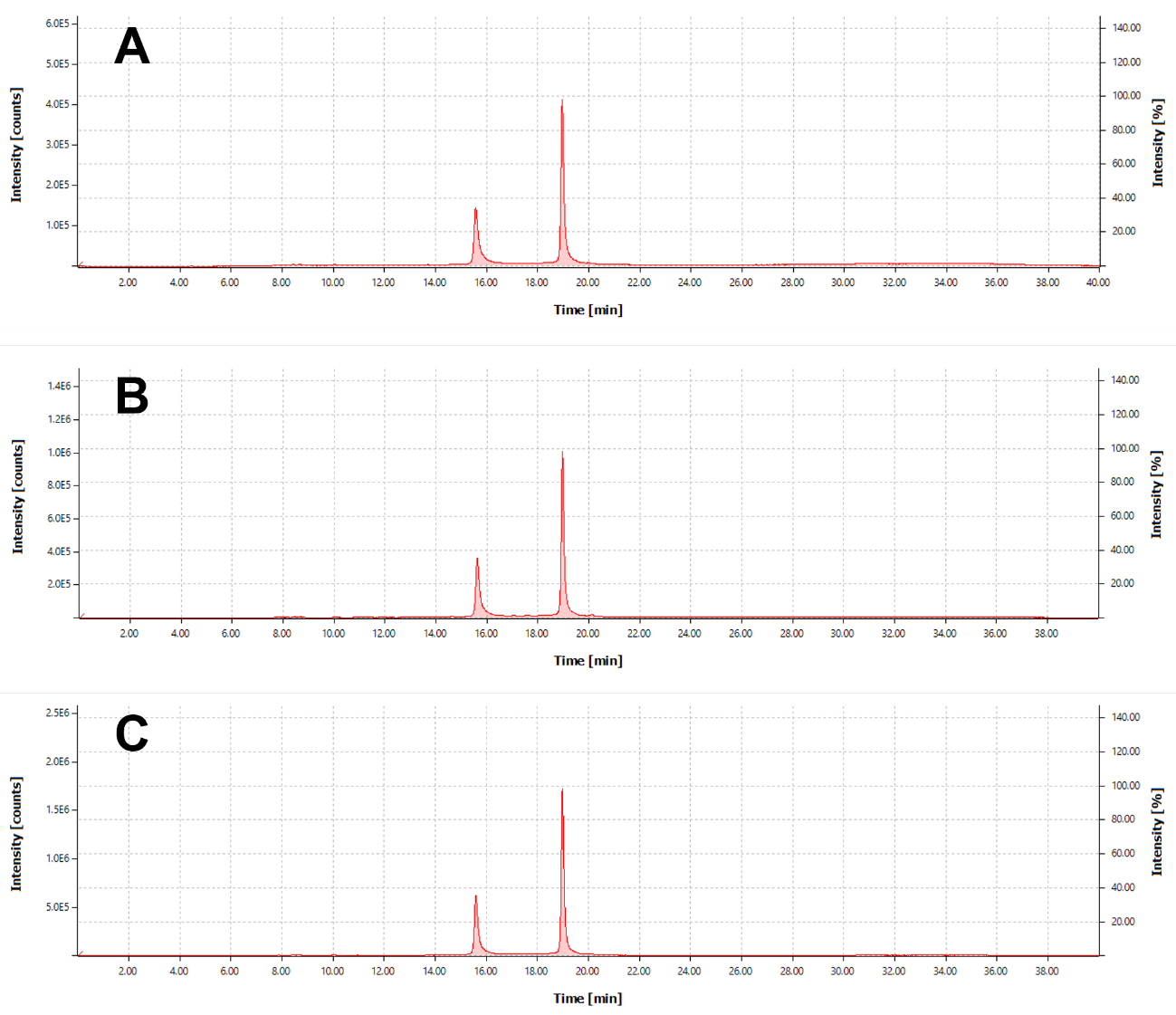


**Figure S-1**. (A) Chromatogram of the molecular standard of punicalagin at 0.1 mg/mL. Peak 1 area: 17413792.13, Peak 2 area: 34762477.23. (B) Chromatogram of the molecular standard of punicalagin at 0.3 mg/mL. Peak 1 area: 40499982.35, Peak 2 area: 81501109.39. (C) Chromatogram of the molecular standard of punicalagin at 0.5 mg/mL. Peak 1 area: 71052585.02, Peak 2 area: 137654991.7.

**Figure S-2**. Graphical representation of the standard line of punicalagin used for the quantification of PGE.

**Figure S-3**. (A) Size distribution of AgNPs-OPT. (B) Zeta potential analysis of AgNPs-OPT. (C) Size distribution of AgNPs-HDD (D) Size distribution of AgNPs-PDI (E) Zeta potential analysis of AgNPs-ZP.

**Figure S-4**. (A) FESEM image of AgNPs-OPT used for elemental mapping (B) EDX analysis of AgNPs-OPT. (C) FESEM image of AgNPs-HDD used for elemental mapping (D) EDX analysis of AgNPs-HDD (E) FESEM image of AgNPs-PDI used for elemental mapping. (F) EDX analysis of AgNPs-PDI. (G) FESEM image of AgNPs-ZP used for elemental mapping. (H) (F) EDX analysis of AgNPs-ZP. Scale bar: 0.5 µm.

**Figure S-5**. (A) FESEM image of treated S. aureus used for elemental mapping. Scale bar: 1 µm. (B) EDX analysis of AgNPs found inside of S. aureus.


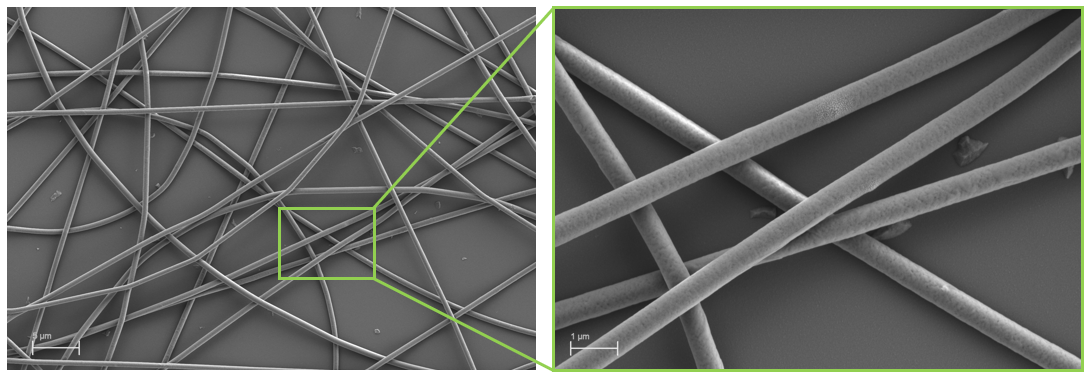


**Figure S-6**. FESEM image (SE2 detector, EHT=1 kV) of the surface of nanofibers synthesized from 3% PEO/15% BSA/0.2% AgNPs-OPT. Scale bar: 1 µm.

**Figure S-7**. (A) FESEM image of nanofibers synthesized from 3% PEO/15% BSA/0.2% AgNPs-OPT used for elemental mapping. (B) EDX analysis of AgNPs found inside of the nanofibers. Scale bar: 1 µm.
